# Occupational History and Health Status Among Older Adults in Ecuador: Evidence from the SABE Survey

**DOI:** 10.3390/ijerph23020210

**Published:** 2026-02-08

**Authors:** Christian F. Juna, Galilea Jarrín, Álvaro Morales, Erika Guerra, Hyojee Joung

**Affiliations:** 1 Health and Care Research Group, Pontifical Catholic University of Ecuador, Quito 170525, Ecuador; gdjarrin@puce.edu.ec (G.J.); eaguerrad@puce.edu.ec (E.G.); 2 Carrera de Enfermería, Instituto Superior Tecnológico ISMAC, Quito 170184, Ecuador; alvarom@tecnologicoismac.edu.ec; 3 Department of Public Health, Graduate School of Public Health, Seoul National University, Seoul 08826, Republic of Korea; hjjoung@snu.ac.kr; 4 Institute of Health and Environment, Seoul National University, Seoul 08826, Republic of Korea

**Keywords:** occupational exposure, older adults, self-rated health, chronic respiratory disease, SABE Ecuador

## Abstract

**Highlights:**

**Public health relevance—How does this work relate to a public health issue?**

- Uses nationally representative SABE data to show how lifetime occupational exposures shape health among older adults in Ecuador.
- Documents that physically demanding and agricultural work is associated with poorer self-rated health and higher chronic respiratory disease, particularly among women.

**Public health significance—Why is this work of significance to public health?**

- Demonstrates that late-life health inequalities in Ecuador partly reflect long-term, accumulated occupational risks in an unequal and largely informal labor market.
- Provides rare, population-based evidence from a middle-income Latin American country, complementing existing data from high-income settings.

**Public health implications—What are the key implications or messages for practitioners, policy makers and/or researchers in public health?**

- Supports incorporating structured occupational history into geriatric assessment and risk stratification in primary care and respiratory services.
- Highlights the need for stronger, gender-sensitive occupational protections—especially in agriculture—to reduce inequities and promote healthy aging.

**Abstract:**

Occupational conditions across the life course may leave a lasting imprint on health in later life, particularly in unequal and largely informal labor markets. This study examined associations between lifetime occupational history and health status among older adults in Ecuador using nationally representative data. We analyzed 5235 participants aged ≥ 60 years from the SABE Ecuador 2009 survey. Occupational history was characterized by economic sector, physical demands, and self-reported exposure to dust, chemicals, heat, and other hazards. Health outcomes included self-rated health, physician-diagnosed diabetes, hypertension, chronic respiratory disease, arthritis, and visual and hearing limitations. We estimated survey-weighted PRs using Poisson regression with robust variance, adjusting for age, sex, education, region, residence, marital status, and household assets, and conducted sex-stratified analyses. Physically demanding work trajectories were associated with a higher prevalence of fair/poor self-rated health (adjusted PR 1.28; 95% CI: 1.10–1.49), with stronger effects in women. Agricultural employment was associated with chronic respiratory disease among women (adjusted PR 1.62; 95% CI: 1.12–2.36), but not men. These findings suggest that long-term occupational exposures contribute to health inequalities in older Ecuadorians and support integrating occupational history into geriatric assessment and strengthening gender-sensitive occupational health protections, particularly in the agricultural sector.

## 1. Introduction

Population aging is accelerating worldwide, including in Latin America, where it is occurring in parallel with persistent social inequality, labor informality and limited social protection [1,2,3]. In this context, health in older adulthood reflects not only biological aging but also the cumulative imprint of social and occupational exposures across the life course [1,2]. Life-course epidemiology and cumulative disadvantage frameworks emphasize that chronic disease and functional decline in later life are shaped by exposures acting in critical periods, chains of risk and accumulation of advantage or disadvantage over time [1,2]. Occupational histories, spanning job type, physical demands and exposure to hazardous environments, are a central yet often underexplored component of these trajectories [3,4,5].

From a social-epidemiological and ecosocial perspective, work is a key arena where social hierarchies and power relations become embodied as differential exposure and vulnerability [3,4,5]. Workers in lower socioeconomic positions are more likely to enter physically demanding, unstable or hazardous jobs, with limited autonomy and weak regulatory protection [3,4,5]. Repeated exposure to dust, fumes, pesticides, biomechanical strain, heat stress and noise has been linked to chronic respiratory disease, musculoskeletal disorders and other adverse outcomes in diverse settings [4,6,7,8,9]. These processes are consistent with the concept of allostatic load, whereby sustained activation of stress pathways produces multisystem “wear-and-tear” that becomes clinically evident in later life [1,7]. At the same time, there is continuing debate about the relative roles of causal effects of work versus health-related selection into and out of jobs, and about the extent to which working conditions versus broader social disadvantage drive late-life inequalities [1,2].

Latin America offers a particularly salient context in which to study these mechanisms. The region is characterized by high levels of informal employment, weak enforcement of occupational safety standards and marked gender and social stratification in the labor market [3,4,5]. Informal and rural workers frequently lack social security, written contracts or access to occupational health services, which increases their vulnerability to unregulated exposures and income instability [3,4,5]. In Ecuador, older adults experience substantial social and health inequalities that reflect combined effects of poverty, territorial disparities and life-course disadvantages [10]. Over the past two decades, the country has implemented health-system and social-protection reforms that expanded public provision and attempted to reduce financial barriers to care; however, coverage of contributory social security remains incomplete, informality in the labor market is still widespread, and important gaps persist in access, quality and continuity of services for older adults, particularly those with long informal or agricultural work histories [3,4,5,10,11]. Despite this context, occupational determinants of these inequalities remain poorly documented at the population level.

Evidence from high-income countries shows that long-term engagement in manual, physically demanding or low-autonomy occupations is associated with steeper declines in lung function, higher multimorbidity and reduced disability-free life expectancy [1,2,6,8,9]. Studies from Latin America have begun to describe the health effects of informal employment and poor job quality, but they have rarely integrated detailed occupational histories or focused on older adults [3,4,5,10]. Controversy persists regarding sex differences: some studies suggest that women may be more susceptible to respiratory and other effects of occupational exposures, while others attribute observed inequalities primarily to differences in job type and unpaid care responsibilities [3,4,5,6,7,9]. In Ecuador, population-based evidence linking lifetime occupational exposures to health outcomes in later life is still scarce.

The Encuesta de Salud, Bienestar y Envejecimiento SABE Ecuador 2009, provides a unique opportunity to address these gaps, as it combines detailed information on health, functioning and sociodemographic conditions with a dedicated occupational history module [11,12]. Using these data, the present study examines the association between lifetime occupational history and health status among adults aged 60 years and older in Ecuador. Specifically, we analyze how economic sector and physically demanding work relate to self-rated health, chronic diseases and sensory limitations, and whether these associations differ by sex. Through this approach, we aim to clarify the potential contribution of long-term occupational exposures to health inequalities in older age and to inform geriatric assessment and occupational health policy in Ecuador and similar settings.

## 2. Materials and Methods

### 2.1. Study Design and Data Source

We conducted a cross-sectional study using microdata from the National Health, Wellbeing and Aging Survey SABE Ecuador 2009, implemented by the National Institute of Statistics and Census (INEC) in collaboration with national institutions following standardized SABE protocols [11,12]. In this context, microdata refer to one record per participant with individual survey responses, rather than aggregated tabulations. Fieldwork was conducted between June and August 2009 in urban and rural areas of the Coastal and Andean regions. A multistage, stratified cluster sampling design was used, with census sectors as primary sampling units and households as secondary units [11,12]. Sampling weights, strata and primary sampling units provided by INEC were incorporated into all analyses to restore national representativeness and obtain valid variance estimates [13,14].

### 2.2. Study Population

The target population comprised community-dwelling adults aged 60 and older residing in private households in the surveyed regions [11,12]. For this analysis, we included respondents with complete information in the core sociodemographic module and in the health and occupational history modules. We excluded observations with missing or implausible data on age, sex or key exposure and outcome variables. The final analytic sample consisted of 5235 older adults (2468 men and 2767 women).

### 2.3. Measures

#### 2.3.1. Health Outcomes

Health outcomes were obtained from the SABE health module [11,12]. Self-rated health was assessed with the question “In general, how would you rate your health?” with five response categories (excellent, good, fair, poor, very poor). For the main analyses, self-rated health was dichotomized as excellent/good versus fair/poor, consistent with prior evidence that this categorization predicts morbidity and mortality [15,16]. Physician-diagnosed chronic conditions were self-reported based on previous diagnoses and included diabetes, hypertension, chronic respiratory disease (e.g., chronic bronchitis, emphysema, chronic obstructive pulmonary disease) and arthritis. Visual and hearing limitations were assessed using self-reported difficulty seeing (with glasses if used) and hearing (with a hearing aid if used) and dichotomized as any versus no difficulty.

#### 2.3.2. Occupational History

Occupational history was derived from the SABE occupational module, which collects retrospective information on lifetime employment [11,12]. We considered: (i) ever having worked versus never having worked; (ii) primary lifetime occupation, classified by economic sector using national adaptations of the International Standard Industrial Classification; and (iii) self-reported exposure during working life to dust, fumes or smoke, chemicals, biological agents, noise and heat. For the main analyses, economic sector was grouped as agriculture/livestock/forestry/fishing (primary sector), manufacturing/industry, commerce/transport and other services. We also created an indicator of physically demanding work based on reported manual labor intensity, prolonged standing and heavy lifting, categorized as higher versus lower physical demand. These indicators were selected because chronic exposure to dust, agrochemicals and physical workload has been associated with respiratory and musculoskeletal outcomes in previous studies [4,6,7,9].

#### 2.3.3. Covariates

Covariates were chosen a priori as potential confounders based on life-course and social-epidemiological frameworks and prior evidence on health inequalities in Latin America and Ecuador [1,2,3,4,5,10]. Sociodemographic variables included age (continuous and categorized as 60–69, 70–79 and ≥80 years), sex, educational attainment (primary or less, secondary, higher), marital status (married vs. not married), ethnicity, region (Coast vs. Highlands) and area of residence (urban vs. rural). A household asset index was constructed from ownership of durable goods and housing characteristics and used as a proxy for socioeconomic position [10,11,12].

### 2.4. Statistical Analysis

We first described the sociodemographic characteristics, health outcomes and occupational histories of the study population using survey-weighted proportions and 95% confidence intervals (CIs). Differences between groups were assessed using Rao–Scott χ^2^ tests, which adjust the Pearson χ^2^ statistic for complex survey designs [13]. To estimate associations between occupational exposures and binary health outcomes, we used Poisson regression with robust variance to obtain prevalence ratios (PRs) and 95% CIs [17,18]. This approach is preferable to logistic regression when outcomes are common, because it directly estimates PRs and avoids overestimation of effect sizes on the odds ratio scale [17].

All models incorporated sampling weights, strata and primary sampling units using standard procedures for complex survey data [14]. Core models were adjusted for age, sex (when not stratified), educational attainment, marital status, region, area of residence and household asset index as potential confounders [1,2,3,4,5,10]. Sex-stratified models were estimated to examine gender differences. In sensitivity analyses, we re-estimated models excluding participants who reported never having worked and tested alternative categorizations of self-rated health. Analyses were conducted using Stata version 18 (StataCorp LLC, College Station, TX, USA; svy commands) and R version 4.3.0 (R Foundation for Statistical Computing, Vienna, Austria; survey package) after data preparation in IBM SPSS Statistics version 29.0 (IBM Corp., Armonk, NY, USA); annotated analysis code can be made available from the corresponding author upon reasonable request.

### 2.5. Ethical Considerations

SABE Ecuador 2009 was conducted by INEC according to national ethical and statistical regulations; details of ethical approval and informed consent procedures have been reported elsewhere [11,12]. The present study is a secondary analysis of anonymized survey microdata and did not involve contact with participants. In accordance with institutional and national guidelines, no additional ethics committee review was required for this re-analysis of de-identified data.

### 2.6. Data Availability and Use of Generative Artificial Intelligence

SABE Ecuador 2009 microdata and documentation are available from INEC upon reasonable request and subject to the institute’s data access policies [11,12]. The analytical code used for the present study (Stata and R scripts) is available from the corresponding author upon reasonable request, to enable replication and extension of the analyses.

A generative artificial intelligence tool (ChatGPT, GPT-5.1 Thinking model, OpenAI; web-based version accessed on 20 December 2025) was used to assist in structuring and editing the English text of the manuscript (e.g., phrasing, organization of sections). All study design decisions, data management, statistical analyses and interpretation of results were carried out by the authors, who reviewed and took full responsibility for the final content. No data, results or graphics were generated by artificial intelligence.

## 3. Results

A total of 5235 older adults were included in the analysis, of whom 2468 were men and 2767 were women. All results are survey-weighted to represent the national population of community-dwelling adults aged 60 years and older in the surveyed regions.

### 3.1. Sample Characteristics

Table 1 summarizes the sociodemographic characteristics of participants by sex. Most men and women were between 60 and 69 years of age, and a large majority had primary education or less. Women were somewhat more likely than men to be not married and to belong to disadvantaged ethnic and socioeconomic groups. Rural residence and lower household asset levels were frequent in both sexes, reflecting the demographic profile of older Ecuadorians captured by SABE Ecuador 2009.

### 3.2. Chronic Conditions and Sensory Limitations

Chronic diseases and sensory limitations were highly prevalent in the study population (Table 2). Hypertension and diabetes were the most reported conditions, followed by arthritis and chronic respiratory disease. Women generally showed higher prevalence of hypertension, arthritis and visual limitations, while men exhibited slightly higher prevalence of hearing difficulty. Fair or poor self-rate health was common in both sexes and was more frequent among participants with multiple chronic conditions and sensory impairments.

### 3.3. Occupational History

Table 3 presents the prevalence of fair/poor self-rated health among older adults according to their main lifetime economic sector, separately for men and women. Older adults with agricultural work histories showed the highest proportion of fair/poor self-rated health in both sexes, followed by those who had worked in commerce/transport and other sectors, while those in manufacturing generally reported lower levels of fair/poor self-rated health. These patterns suggest that primary-sector work is associated with a greater burden of perceived ill-health in later life, particularly among women, and are consistent with the sector-specific associations observed in the multivariable models.

### 3.4. Associations Between Occupational History and Health Outcomes

Table 4 presents the associations between occupational exposures and health outcomes. In models adjusted for age, education, marital status, region, area of residence and household assets, physically demanding work trajectories were associated with a higher prevalence of fair or poor self-rated health compared with less physically demanding trajectories (adjusted PR 1.28; 95% CI: 1.10–1.49). This association was stronger among women than men in sex-stratified analyses.

Older adults with agricultural work histories had the highest prevalence of fair or poor self-rated health, followed by those employed in manufacturing and other sectors. Agricultural employment was also associated with chronic respiratory disease among women (adjusted PR 1.62; 95% CI: 1.12–2.36), whereas the corresponding estimate among men was smaller in magnitude and not statistically significant. Associations between occupational history and other chronic conditions (diabetes, hypertension, arthritis) were weaker and less consistent.

Sensitivity analyses excluding participants who had never worked yielded similar results for the main associations between physically demanding trajectories, agricultural work and health outcomes. Alternative categorizations of self-rated health did not materially change the direction or interpretation of the findings.

## 4. Discussion

In this nationally representative sample of older adults in Ecuador, lifetime occupational history was strongly associated with health status in later life. Older adults with physically demanding work trajectories and those with agricultural work histories were more likely to report fair or poor self-rated health and, among women, a higher prevalence of chronic respiratory disease. These findings are consistent with our working hypothesis that accumulated occupational exposures across the life course contribute to health inequalities in older age and that these effects are particularly pronounced in the primary sector and among women.

Our results align with life-course and cumulative disadvantage frameworks, which posit that repeated exposure to adverse working and living conditions can become biologically embedded and manifest as chronic disease and functional decline in older adulthood [1,2]. Manual, strenuous and low-autonomy occupations have been associated with steeper declines in functional capacity, reduced disability-free life expectancy and greater multimorbidity in high-income settings [1,2]. The elevated prevalence of poor self-rated health and respiratory morbidity among older adults with physically demanding and agricultural trajectories observed here is compatible with these life-course models and extends previous Latin American evidence on informal and precarious work and health inequalities, refs. [3,4,5,10] indicating that similar processes of cumulative occupational disadvantage operate in a middle-income context.

The associations between agricultural work and chronic respiratory disease, particularly among women, are also consistent with prior evidence on occupational respiratory risks. Chronic exposure to organic dusts, endotoxins, agrochemicals and other airborne irritants in agricultural settings has been linked to chronic obstructive pulmonary disease and related outcomes [6,7,9]. These exposures may be intensified by limited access to personal protective equipment and weak enforcement of occupational safety standards [3,4,5,19,20]. The stronger association between agricultural work and chronic respiratory disease observed among women may reflect the interaction of social and biological mechanisms. In many rural Latin American settings, women combine physically demanding agricultural activities with unpaid domestic and care work, leading to longer working days, limited recovery time and cumulative strain across the life course [3,4,5,10]. Gendered task allocation can also place women in specific roles (e.g., handling agrochemicals or working in enclosed spaces) with high exposure and lower access to personal protective equipment or occupational health services [3,4,5,6,7,9]. At the same time, some evidence suggests sex differences in susceptibility to airway inflammation and lung-function decline in response to environmental and occupational exposures, which could amplify the respiratory impact of similar exposure levels in women compared with men [6,7,9]. These interacting social and biological processes may help explain the stronger associations we observed in women with agricultural work histories.

Our findings contribute to a broader understanding of how informal and precarious employment shapes health in Latin America. Studies from the region have shown that informal employment is associated with poorer self-rated health and other adverse outcomes among working-age adults, particularly in contexts of weak social protection and high labor informality [3,4,5]. However, relatively few analyses have focused on older adults or incorporated detailed occupational histories that span the working life [3,4,5,10]. By exploiting the occupational module of SABE Ecuador 2009, this study extends the literature by documenting that inequalities in late-life health in Ecuador are, at least in part, a legacy of long-term exposure to physically demanding and hazardous work conditions [10,11,12]. This reinforces the idea that current health inequalities among older people cannot be understood without reference to their labor trajectories and the structural features of the labor market in which they worked.

The use of self-rated health as a key outcome is also relevant for public health. Self-rated health is a simple, widely used measure that integrates physical, mental and social dimensions of health and consistently predicts mortality and other hard outcomes in older adults [15,16]. The fact that physically demanding and agricultural work trajectories remained associated with fair or poor self-rated health after adjustment for socioeconomic indicators suggests that these occupational effects are not fully explained by measured social position and may capture additional, unobserved dimensions of cumulative risk [1,2,3,10]. Similarly, the sex-specific association between agricultural work and chronic respiratory disease supports the hypothesis that certain occupational exposures exert independent effects on specific disease outcomes [6,7,9].

This study has several strengths. First, it uses a nationally representative survey with a dedicated occupational history module, allowing us to examine lifetime occupational exposures rather than only current or last job [11,12]. Second, we applied complex survey methods and used Poisson regression with robust variance to estimate PRs, an approach appropriate for common outcomes and cross-sectional data [13,14,17,18]. Third, we considered a range of health outcomes, including self-rated health, chronic conditions and sensory limitations, and examined sex-stratified models to explore gender differences. The consistency of our findings across sensitivity analyses further supports their robustness.

However, some limitations must be acknowledged. The cross-sectional design precludes firm causal inference; although occupational exposures precede late-life health, reverse causation and health-related selection into or out of specific jobs cannot be ruled out [1,2]. Health outcomes and occupational exposures were self-reported and may be subject to recall and reporting biases. Nevertheless, self-rated health and self-reported chronic conditions have acceptable validity as predictors of morbidity and mortality in older populations [15,16]. In contrast, occupational exposures such as dust, chemicals, smoke and heat were assessed retrospectively and in broad categories, without standardized measures of duration or intensity; this reliance on recall and coarse indicators may have led to exposure misclassification, particularly for long and complex work trajectories, and likely resulted in non-differential bias that would attenuate rather than exaggerate the true associations. The classification of economic sector is relatively coarse and does not capture the full complexity of job tasks, intensity and duration of exposure, or job transitions across the life course, which may lead to non-differential misclassification and attenuation of associations [6,7,9]. Survivor bias is also possible, as individuals with the highest exposure and vulnerability may have died before the survey, potentially underestimating the true magnitude of occupational effects. A further limitation is the age of the data: SABE Ecuador was conducted in 2009–2010, so the labor market, social policies and health system have undergone important changes since then. However, to our knowledge, SABE 2009–2010 remains the only nationally representative survey of older adults in Ecuador that includes both detailed occupational histories and health outcomes [11,12], and subsequent analyses of aging and social inequalities in the country continue to rely on this source [10]. We therefore interpret our results as documenting baseline associations between lifetime occupational trajectories and health in older age, rather than providing up-to-date prevalence estimates; new rounds of nationally representative aging surveys will be needed to update and extend these findings.

The results of this study have important implications for research, clinical practice and public health. For clinicians and geriatric services, our findings support the systematic integration of structured occupational histories into the routine assessment of older adults, particularly those with respiratory symptoms or multiple chronic conditions, to improve risk stratification and target management. From a public health perspective, the results highlight the need to strengthen regulation, surveillance and primary prevention in high-risk sectors such as agriculture, with explicit attention to gender, informality and life-course perspectives [3,4,5,19,20]. This includes improving occupational health services, monitoring work-related respiratory disease and integrating occupational exposures into national strategies for noncommunicable diseases and healthy aging. For social protection and labor policy, the evidence suggests that improving working conditions, expanding coverage of social security and recognizing unpaid care work are not only labor rights issues but also key public health interventions that may yield long-term benefits for healthy aging and the reduction of health inequalities in Ecuador [3,4,5,10].

Future research should build on these findings using longitudinal designs, more granular and objective exposure assessment (e.g., duration of exposure, job–exposure matrices or linkage with administrative data) and linkage with clinical measurements such as spirometry for respiratory function to clarify temporal relationships, causal pathways and the biological mechanisms linking work trajectories to health in older age. Prospective cohort studies and follow-up surveys could clarify temporal relationships and disentangle the relative contributions of occupational exposures, social position and health selection [1,2]. Incorporating biomarkers of inflammation, lung function and other physiological indicators would allow a more detailed examination of biological pathways linking work and aging [6,7,8,9]. Finally, comparative analyses across countries with different labor-market regimes and social protection systems could illuminate how institutional contexts moderate the long-term health impact of occupational histories and help identify policy levers that are most effective in reducing occupationally driven health inequalities in older age [3,4,5,10,11].

In summary, this study shows that lifetime occupational trajectories, especially physically demanding and agricultural work, are associated with poorer health among older adults in Ecuador, with stronger effects observed in women. These results underscore the need to view healthy aging as the cumulative outcome of social and occupational conditions over the course of life and to design interventions that protect workers long before they reach old age, thereby promoting more equitable health trajectories in later life [1,2,3,4,5,19,20].

## 5. Conclusions

This study shows that lifetime occupational trajectories, particularly physically demanding work and employment in the agricultural sector, are associated with poorer health among older adults in Ecuador. Older people with these work histories were more likely to report fair or poor self-rated health, and women with agricultural work histories had a higher prevalence of chronic respiratory disease.

These findings suggest that late-life health inequalities in Ecuador are shaped not only by current access to care and material living conditions but also by cumulative social and occupational disadvantages across the life course, such as early-life poverty, limited educational opportunities, long periods of informal and precarious employment, and gendered divisions of paid and unpaid work, within an unequal and largely informal labor market [3,4,5,12]. Integrating structured occupational histories into geriatric assessment and strengthening occupational health regulation, surveillance and primary prevention, especially in agriculture and other high-risk sectors, together with policies to improve job quality, expand social protection and reduce territorial and gender inequalities, may contribute to healthier and more equitable aging.

Future research should use longitudinal designs and more detailed exposure assessment to clarify causal pathways, explore biological mechanisms and evaluate how different labor-market and social protection regimes shape the long-term health consequences of work.

## Figures and Tables

**Table 1:** Sociodemographic characteristics of older adults by sex (SABE Ecuador 2009).

	Men (n = 2468)	Women (n = 2767)
Age group (% *)		
60–69 years	72.2	73.5
70–79 years	21.9	21.6
≥80 years	5.9	4.9
Educational level (% *)		
Primary or less	78.5	77.2
Secondary	17.6	18.1
Higher education	3.9	4.7
Marital status (% *)		
Married	74.8	72.9
Not married	25.2	27.1
Ethnicity (% *)		
Mestizo	74.5	73.7
Indigenous	21.4	20.4
Afro-Ecuadorian	4.1	5.9

* % Weighted percentages accounting for the complex survey design (strata, clusters and sampling weights).

**Table 2:** Prevalence of chronic conditions and sensory limitations among older adults.

Chronic Condition	Men (% *)	Women (% *)
Diabetes	22.6	25.7
Hypertension	26.3	25.7
Chronic respiratory disease	35.0	36.7
Poor vision	35.6	39.7
Poor hearing	38.7	38.1

* % Weighted percentages accounting for the complex survey design (strata, clusters and sampling weights).

**Table 3:** Prevalence of fair/poor self-rated health by lifetime economic sector and sex.

Economic Sector	Men (% *)	Women (% *)
Agriculture	31.5	32.1
Manufacturing	16.7	22.1
Commerce/Transport	20.5	25.5
Other sectors	19.9	21.4

* % Weighted percentages accounting for the complex survey design (strata, clusters and sampling weights).

**Table 4:** Association between agricultural work and chronic respiratory disease.

Sex	Adjusted PR *	95% CI
Men	1.14	0.78–1.66
Women	1.62	1.12–2.36

* Adjusted for age, educational level, marital status, region, area of residence and household asset index.

## Data Availability

This study is based on secondary analysis of microdata from the SABE Ecuador 2009 survey, collected by the National Institute of Statistics and Census (INEC). The SABE Ecuador 2009 dataset and accompanying documentation are available in the INEC microdata catalog under “Encuesta de Salud, Bienestar y Envejecimiento 2009 (SABE 2009)” at: https://anda.inec.gob.ec/anda/index.php/catalog/292 (accessed on 8 January 2025). No new data were created or collected specifically for this study. The annotated analysis code (Stata and R scripts) used to generate the results is available from the corresponding author upon reasonable request.

## Abbreviations

The following abbreviations are used in this manuscript:

SABE	Encuesta de Salud, Bienestar y Envejecimiento
INEC	Instituto Nacional de Estadística y Censos
PR	Prevalence Ratio
CI	Confidence Interval
